# The Biochemical and Molecular Analysis of Changes in Melanogenesis Induced by UVA-Activated Fluoroquinolones—In Vitro Study on Human Normal Melanocytes

**DOI:** 10.3390/cells10112900

**Published:** 2021-10-27

**Authors:** Justyna Kowalska, Klaudia Banach, Artur Beberok, Jakub Rok, Zuzanna Rzepka, Dorota Wrześniok

**Affiliations:** Department of Pharmaceutical Chemistry, Faculty of Pharmaceutical Sciences in Sosnowiec, Medical University of Silesia, Katowice, 41-200 Sosnowiec, Poland; jkowalska@sum.edu.pl (J.K.); kbanach@sum.edu.pl (K.B.); abeberok@sum.edu.pl (A.B.); jrok@sum.edu.pl (J.R.); zrzepka@sum.edu.pl (Z.R.)

**Keywords:** moxifloxacin, lomefloxacin, melanin, tyrosinase, microphthalmia-associated transcription factor, melanogenesis

## Abstract

Fluoroquinolones cause phototoxic reactions, manifested as different types of skin lesions, including hyperpigmentation. The disturbances of melanogenesis indicate that fluoroquinolones may affect cellular processes in melanocytes. It has been reported that these antibiotics may bind with melanin and accumulate in pigmented cells. The study aimed to examine the changes in melanogenesis in human normal melanocytes exposed to UVA radiation and treated with lomefloxacin and moxifloxacin, the most and the least fluoroquinolone, respectively. The obtained results demonstrated that both tested fluoroquinolones inhibited melanogenesis through a decrease in tyrosinase activity and down-regulation of tyrosinase and microphthalmia-associated transcription factor production. Only lomefloxacin potentiated UVA-induced melanogenesis. Under UVA irradiation lomefloxacin significantly enhanced melanin content and tyrosinase activity in melanocytes, although the drug did not cause an increased expression of tyrosinase or microphthalmia-associated transcription factor. The current studies revealed that phototoxic activity of fluoroquinolones is associated with alterations in the melanogenesis process. The difference in phototoxic potential of fluoroquinolones derivatives may be connected with various effects on UVA-induced events at a cellular level.

## 1. Introduction

Fluoroquinolones (FQs) are a large group of synthetic antibiotics with broad-spectrum bactericidal activity. They are indicated for the treatment wide range of infectious diseases, including genitourinary infections, respiratory infections, gastrointestinal infections, sexually transmitted diseases, and skin infections [1,2]. FQs are active against Gram-positive and -negative bacteria, that is attribbuted to inhibition of topoisomerase II (DNA gyrase) and IV—two enzymes essential for DNA replication in bacteria [3,4]. Moreover, FQs have been shown to exhibit antifungal [5,6] and antiviral activity [7]. Taking into account that topoisomerases are also important for human cells, the antitumor activity of FQs was also analyzed. The in vitro studies indicated the anticancer potential of FQs against melanoma [8,9,10] and cervical, lung, urinary tract, and pancreatic cancers [11,12]. Due to the possibility of using FQs for new indications, it is important to improve knowledge about the safety profile of these drugs.

**Figure 1:**
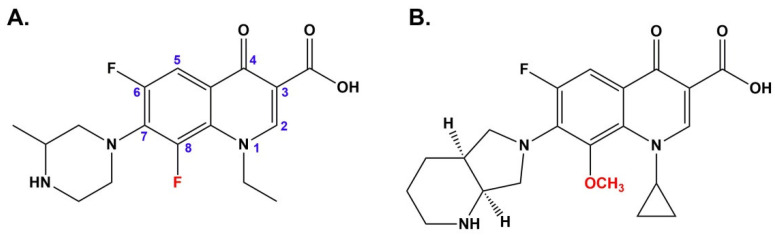
Chemical structures of lomefloxacin (**A**) and moxifloxacin (**B**).

Adverse effects associated with fluoroquinolones therapy include gastrointestinal, central nervous system, and dermatological reactions. The most common skin lesions reported during FQs therapy are rash and pruritus. In the case of photosensitivity, phototoxicity is more frequent than photoallergy [13]. Clinical manifestation of FQs-induced phototoxicity is a sunburn-like reaction resulting in hyperpigmentation [14]. There are differences in phototoxic potential among the FQs and they are related to the chemical structure. It has been demonstrated that FQs with a fluorine substitution at position eight are unstable under UVA light irradiation, in contrast to 8-methoxy derivatives [15]. Moreover, Marutani et al. [16] have indicated that the phototoxicity of FQs containing fluorine atom at position eight is stronger than drugs with an 8-methoxyl substituent. Under UVA radiation 8-fluorinated analogs undergo selective dehalogenation at position eight that is associated with the generation of aryl cations. Due to the carbene features, aryl cations are very reactive and interact with i.a. oxygen. The reaction of aryl cations with oxygen leads to the formation of reactive oxygen species, in particular hydrogen peroxide and hydroxyl radicals responsible for cell damage. In contrast, FQs with a methoxy group at the 8 position are resistant to UVA radiation, and they are not converted to reactive aryl cations [16,17,18]. Therefore, lomefloxacin is considered the most phototoxic FQ and moxifloxacin for the least (Figure 1) [14].

Cutaneous side effects of FQs may result from their accumulation in skin cells. Previously, we showed that lomefloxacin, ciprofloxacin, sparfloxacin, norfloxacin, and moxifloxacin bound to melanin biopolymer [19,20]. FQ-derivatives differ in a number of binding sites on melanin that may lead to differences in pharmacokinetics and skin toxicity between drugs of this antibiotic class. Among others, the least phototoxic moxifloxacin is characterized by the lowest number of binding sites on melanin.

Melanocytes are highly differentiated cells, responsible for melanin production. In the skin, melanocytes are located in the basal layer of the epidermis and hair bulb. Moreover, these cells reside in the iris, inner ear, heart, and nervous system. Melanocytes exhibit a dendritic morphology and possess special membrane-bound organelles, named melanosomes, in the cytoplasm. The functions of melanosomes are synthesis, storage, and transport of melanin [21,22,23].

The purpose of this study was to examine the effect of lomefloxacin and moxifloxacin, the most and the least phototoxic FQ, on melanogenesis in normal human epidermal melanocytes exposed to UVA radiation. The studies included the evaluation of the content of melanin, tyrosinase activity and expression of tyrosinase, and microphthalmia-associated transcription factor (MITF).

## 2. Materials and Methods

### 2.1. Chemicals

Moxifloxacin hydrochloride (Avelox^TM^ solution for i.v. use containing 400 mg of moxifloxacin per 250 mL in 0.8% saline) was purchased from Bayer Healthcare Pharmaceuticals Inc. (Berlin, Germany). Lomefloxacin hydrochloride, synthetic melanin, 3,4-dihydroxy-l-phenylalanine (l-DOPA), Dulbecco’s phosphate-buffered saline (DPBS) with CaCl_2_ and MgCl_2_, phosphate buffered saline (PBS), amphotericin B, penicillin G, SIGMAFAST™ Protease Inhibitor Coctail Tablet, and Phosphatase Inhibitor Coctail 3 were obtained from Sigma–Aldrich Inc. (St. Louis, MO, USA). Trypsin/EDTA solution, growth medium M-254, and a human melanocyte growth supplement-2 (HMGS-2) were purchased from Cascade Biologics/Gibco (Carlsbad, CA, USA). Neomycin sulfate was obtained from Amara (Kraków, Poland). Pierce BCA Protein Assay Kit and ECL Western Blotting Substrate were purchased from Thermo Fisher Scientific (Waltham, MA, USA).

Immunoblot analysis was made by using: Tyrosinase (T311) Mouse mAb from Thermo Fisher Scientific (Waltham, MA, USA), MITF (D5G7V) Rabbit mAb, GAPDH (14C10) Rabbit mAb, from Cell Signaling (Danvers, MA, USA), Anti-Rabbit IgG (A154), Anti-Mouse IgG, Tween-20, RIPA Buffer and PVDF membranes from Sigma–Aldrich Inc. (St. Louis, MO, USA), and Color Prestained Protein Standard from New England Biolabs (Hitchin, UK).

RT-PCR analysis was performed using: SensiFAST™ SYBR No-ROX kit from Bioline (London, UK), KiCqStart SYBRGreen Primers (TYR, MITF, GAPDH) from Sigma–Aldrich Inc. (St. Louis, MO, USA), and TRIZOL reagent from Thermo Fisher Scientific (Waltham, MA, USA).

### 2.2. Cell Culture

Normal human epidermal melanocytes, dark pigmented (HEMn-DP) were purchased from Cascade Biologics (UK). The cells were cultured in an M-254 medium at 37 °C in 5% CO_2_. The medium was supplemented with HMGS-2 as well as neomycin (10 μg/mL), penicillin (100 U/mL), and amphotericin B (0.25 μg/mL). All experiments were performed using cells in passages 5–9.

### 2.3. Treatment of Cells with Fluoroquinolones and UVA Irradiation

Melanocytes were seeded into Petri dishes (1,000,000 cells/dish) into 96-well microplate (2500 cells/well) in a supplemented growth medium and incubated at 37 °C and 5% CO_2_ for 48 h. Then the medium was removed and the cells were treated with fluoroquinolones for 24 h. After exchanging the medium/ drugs solutions with DPBS cells were irradiated with 365 nm UVA (1.3 J/cm^2^) for 30 min using a filtered lamp BVL-8.LM (Vilber Lourmat, Collégien, France). The nonirradiated cells were incubated in the dark at 37 °C and 5% CO_2_. After, DPBS was replaced by the culture medium in all samples and melanocytes were cultured for the next 4 h, 8 h, or 24 h.

### 2.4. Preparation of Cell Lysates

For the analysis of melanization process, melanocytes were trypsynised 24 h after the irradiation. Next, cells were counted using NucleoCounter NC-3000 fluorescence image cytometer, centrifuged, and suspended in a lysis buffer, containing PBS, phosphatase, and protease inhibitors.

For the Western blot analysis, 24 h after the irradiation melanocytes were lysed in RIPA buffer with phosphatase and protease inhibitors. Afterward lysates were centrifuged at 12,000 rpm for 10 min at 4 °C to the melanin separation.

Total protein concentrations were quantitated spectrophotometrically (Denovix DS-11) using Pierce™ BCA Protein Assay Kit (Thermo Scientific, Waltham, MA, USA).

### 2.5. Analysis of Melanization Process

Melanin content in melanocytes was determined according to the previously described method [24]. Cell lysates were heated with 1 M NaOH at 80 °C for 1 h and centrifuged at 16,000× *g* for 20 min. The supernatants’ absorbance was measured at 405 nm. Tyrosinase activity in cells was estimated by a spectrophotometric assay based on oxidation l-DOPA to DOPAchrome [25]. Melanocytes lysates and 2 mg/mL l-DOPA were placed in a 96-well plate (100 µL and 40 µL per well, respectively). Absorbance was measured every 10 min for 90 min at 475 nm at 37 °C. The melanin content and enzyme activity were expressed as the percentage of the control (untreated cells).

### 2.6. Western Blot Analysis

Protein extracts (20 µg/lane) were subjected to 10% SDS- polyacrylamide gel electrophoresis and transferred to PVDF membranes. After 1 h of blocking with 5% non-fat milk in TBS-T (0.1% Tris-buffered saline supplemented with Tween 20), membranes were incubated with following primary antibodies overnight at 4 °C: rabbit anti-GAPDH (1:1000), rabbit anti-MITF (1:500), and mouse anti-TYR (1:70). Subsequently, the membranes were washed and incubated with a horseradish peroxidase-conjugated secondary antibody at room temperature for 1.5 h and detected by enhanced chemiluminescence (ECL) kit. The binding antibodies were visualized using via G:Box Chemi-XT4 Imaging System.

### 2.7. Real-Time Quantitative PCR

Melanocytes were harvested after 4 h and 8 h of incubation since the irradiation and were treated with TRIZOL reagent to RNA extraction. RNA extracts were purified from melanin using OneStep™ PCR Inhibitor Removal Kit (Zymo Research, Irvine, CA, USA). The RNA concentrations and quality were determined spectrophotometrically (Denovix DS-11). qRT-PCR analysis was performed on a real-time fluorescence quantitative PCR instrument (LightCycler^®^ 96 Instrument, Roche Applied Science, Madison, WI, USA) using SensiFAST™ SYBR No-ROX kit (Bioline, London, UK) and primer pair specific for GAPDH, MITF, TYR (nucleotide sequences of primers are described in Table 1). The PCR cycling conditions were 10 min at 45 °C, 2 min at 95 °C followed by 45 cycles of 5 s at 95 °C, 10 s at 60 °C, 1 min at 72 °C, and final extension for 10 min at 72 °C. Quantitation of gene expression was determined by calculating 2^−∆∆Ct^ values.

### 2.8. Statistical Analysis

Data were presented as mean values ± SD of three separate experiments performed in triplicate. The results were analyzed statistically by means of one-way ANOVA (the influence of UVA radiation or FQs) and two-way ANOVA (the influence of UVA radiation and FQs) as well as Dunnett’s and Tukey’s multiple comparison tests, using GraphPad Prism 6.01 Software (GraphPad Software, San Diego, CA, USA). *p*-value < 0.05 was considered of statistically significant difference.

## 3. Results

### 3.1. The Influence of Fluoroquinolones and UVA Radiation on Cell Viability

Taking into account the previous observed impact of lomefloxacin and moxifloxacin, alone and plus UVA radiation, on melanocytes viability [26], in this study we used lomefloxacin in concentrations 0.005 mM, 0.05 mM, 0.5 mM, and moxifloxacin in concentrations 0.01 mM, 0.1 mM, 1.0 mM.

**Figure 2:**
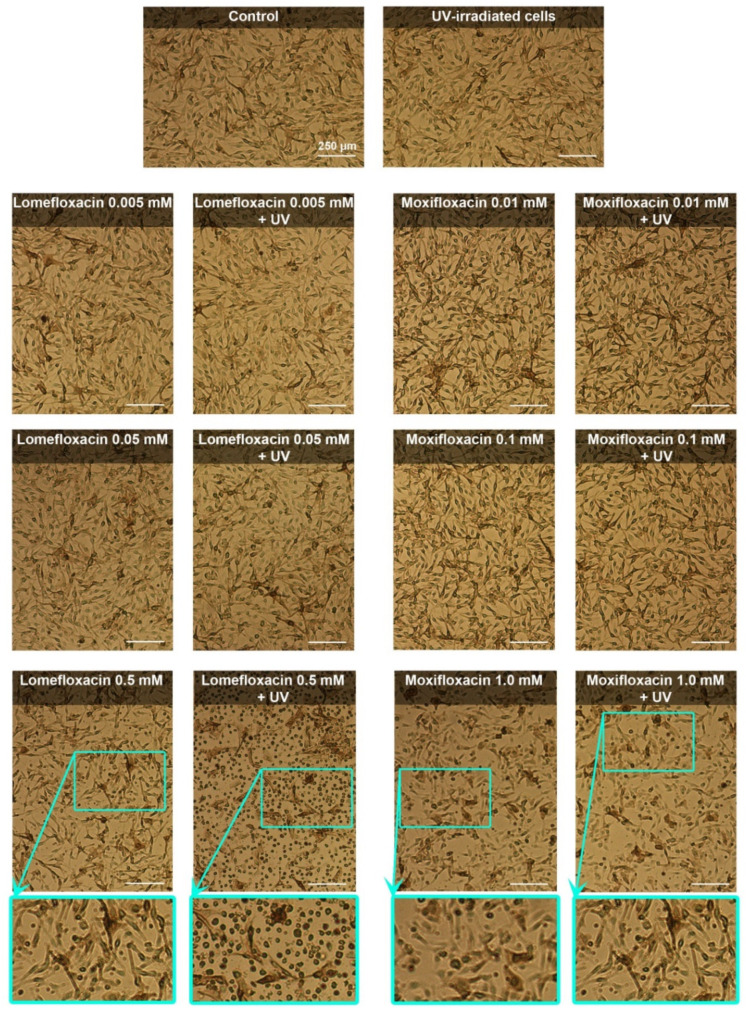
Cytotoxic and phototoxic effects of fluoroquinolones. The melanocytes were treated with lomefloxacin in concentrations of 0.005 mM, 0.05 mM, and 0.5 mM and moxifloxacin in concentrations 0.01 mM, 0.1 mM, and 1.0 mM. Scale bar = 250 µm.

The morphology of melanocytes was analyzed by the use of an inverted microscope Eclipse TS-100-F (Nikon, Tokyo, Japan). As demonstrated in Figure 2, UVA radiation alone did not cause pronounced changes in cell morphology, compared with the control. We observed a decrease in cell number and loss of typical shape in the case of moxifloxacin in concentration 1.0 mM. Irradiation for 30 min. did not enhance the cytotoxic effect of moxifloxacin. Treatment with lomefloxacin in concentration 0.5 mM resulted in a reduction of melanocytes number and length of cellular extensions. The most noticeable alterations in cell morphology were caused by simultaneous exposure to UVA radiation and lomefloxacin in concentration 0.5 mM. UVA irradiation potentiated the cytotoxic potential of lomefloxacin leading to an increase in the number of spherical cells.

**Figure 3:**
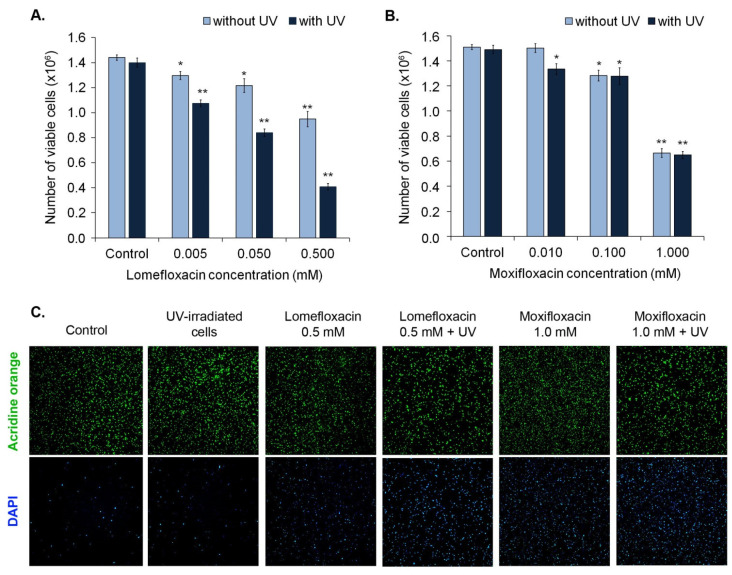
Impact of fluoroquinolones on melanocytes viability. The cells were exposed to lomefloxacin in concentrations of 0.005 mM, 0.05 mM, and 0.5 mM (**A**) and moxifloxacin in concentrations of 0.01 mM, 0.1 mM, and 1.0 mM (**B**). Cell number was determined after 24 h since the irradiation. Bar graph represents mean number of cells per dish ± SD from three independent experiments. * *p* < 0.05, ** *p* < 0.01 vs control. (**C**). Representative images were obtained using NucleoCounter NC-3000 fluorescence image cytometer. Acridine orange stained all cells in the sample and DAPI stained the dead cells.

Cell viability was determined by image cytometric analysis using viability and cell count assay. As shown in Figure 3, the exposure of melanocytes to both moxifloxacin and lomefloxacin led to the decrease in the number of viable (AO-positive, DAPI-negative) cells in a concentration-dependent manner. However, UVA radiation potentiated only the impact of lomefloxacin only on the viability of cells.

### 3.2. The Influence of Fluoroquinolones and UVA Radiation on Melanin Content in Melanocytes

**Figure 4:**
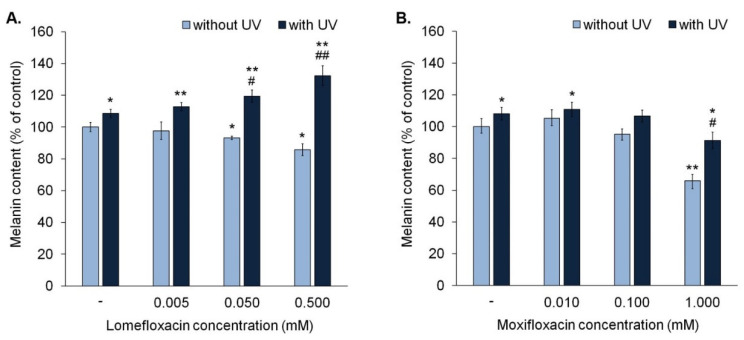
Fluoroquinolones alone decrease melanin content in melanocytes. Lomefloxacin potentiates the impact of UVA radiation (1.3 J/cm^2^) on melanin content. The cells were exposed to lomefloxacin in concentrations of 0.005 mM, 0.05 mM, and 0.5 mM (**A**) and moxifloxacin in concentrations of 0.01 mM, 0.1 mM, and 1.0 mM (**B**). Data were presented as % of the control. Bar graph represents mean ± SD from three independent experiments. * *p* < 0.05, ** *p* < 0.01 vs. control; # *p* < 0.05, ## *p* < 0.01 vs. non-treated cells exposed to UVA radiation.

The obtained data revealed that lomefloxacin in concentrations 0.05 mM and 0.5 mM significantly decreased the melanin content by about 7% and 14%, respectively (Figure 4A), whereas moxifloxacin only in concentration 1.0 mM caused a significant decrease in melanin content by 34% (Figure 4B).

UVA irradiation of melanocytes caused an increase in melanin content by about 8%. It was observed that simultaneous exposure to UVA radiation and lomefloxacin in entire range concentrations resulted in a rise of melanin content in cells (from 13% to 32%), when compared with the control. Moreover, the irradiation of melanocytes treated with lomefloxacin in concentrations 0.05 mM and 0.5 mM caused a significant increase in melanin content, compared with the non-treated cells exposed to UVA radiation.

In the case of moxifloxacin, we observed that simultaneous exposure to UVA radiation and moxifloxacin in concentration 0.01 mM resulted in a significant increase in melanin content by 11%, compared with the control. However, the irradiation of melanocytes treated with moxifloxacin in all tested concentrations did not cause a rise in melanin content compared with non-treated cells exposed to UVA radiation.

### 3.3. The Influence of Fluoroquinolones and UVA Radiation on Activity of Tyrosinase

**Figure 5:**
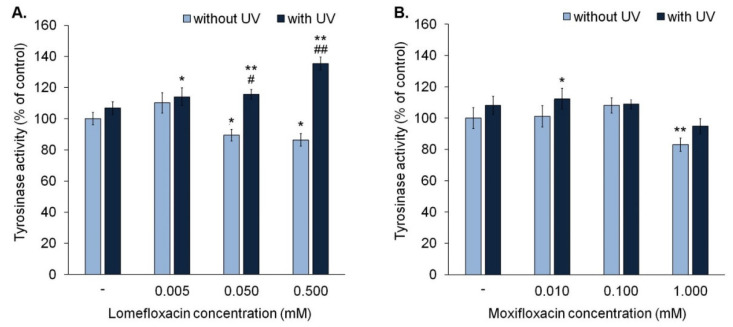
Lomefloxacin magnifies the effect of UVA radiation (1.3 J/cm^2^) on tyrosinase activity. The cells were exposed to lomefloxacin in concentrations of 0.005 mM, 0.05 mM, and 0.5 mM (**A**) and moxifloxacin in concentrations 0.01 mM, 0.1 mM, and 1.0 mM (**B**). Data were presented as % of the control. Bar graph represents mean ± SD from three independent experiments. * *p* < 0.05, ** *p* < 0.01 vs. control; # *p* < 0.05, ## *p* < 0.01 vs. non-treated cells exposed to UVA radiation.

The analysis of tyrosinase activity indicated that lomefloxacin in concentrations 0.05 mM and 0.5 mM suppressed tyrosinase activity to approximately 90% and 86% (Figure 5A), respectively, whereas moxifloxacin (1.0 mM) decreased the activity of tyrosinase by about 17% only in the highest tested concentration (Figure 5B).

We observed that exposure to UVA radiation for 30 min is not associated with a significant increase in the activity of tyrosinase. In the case of melanocytes exposed to UVA radiation and lomefloxacin in all tested concentrations, tyrosinase activity increased significantly by about 14–35%, compared with controls. The obtained data showed that lomefloxacin in concentrations of 0.05 mM and 0.5 mM magnified the impact of UVA radiation on tyrosine activity. An increase in enzyme activity in melanocytes exposed to UVA radiation and lomefloxacin (by about 8–29%) was observed, compared to non-treated cells exposed to UVA radiation. Moreover, in cells exposed to UVA radiation and lomefloxacin in concentrations of 0.05 mM and 0.5 mM, tyrosinase activity was significantly greater than in cells exposed to the drug alone (by about 26% and 49%, respectively). In the case of moxifloxacin, a significant increase of tyrosinase activity by about 12% was observed in cells exposed to UVA radiation and moxifloxacin in concentration 0.01 mM.

In contrast with lomefloxacin, moxifloxacin did not potentiate the influence of UVA radiation on tyrosinase activity. In all irradiated samples, activity of tyrosinase was similar. The significant difference in tyrosinase activity between irradiated and non-irradiated cells was noticed only in the case of the exposure to and moxifloxacin in concentration of 1.00 mM (by 11%).

### 3.4. The Influence of Fluoroquinolones and UVA Radiation on Expression of Tyrosinase and MI-Crophthalmia-Associated Transcription Factor

In order to evaluate the impact of fluoroquinolones on tyrosinase and microphthalmia-associated transcription factor expression in normal human melanocytes, cells were treated with lomefloxacin in concentration of 0.05 mM and moxifloxacin in concentration of 0.1 mM.

**Figure 6:**
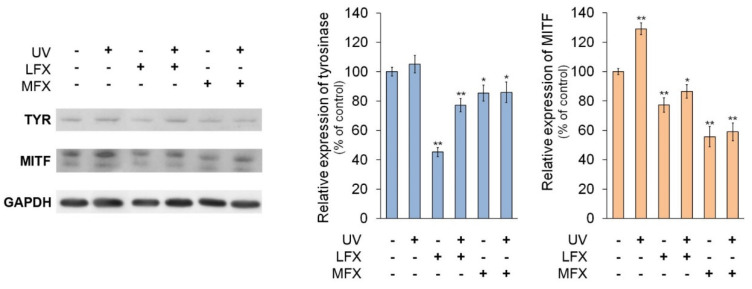
Fluoroquinolones alone and plus UVA radiation modify the expression of tyrosinase and microphthalmia-associated transcription factor (MITF) at the protein level in melanocytes. The cells were exposed to lomefloxacin (LFX) in concentration of 0.05 mM and moxifloxacin (MFX) in concentration of 0.1 mM. The protein levels of tyrosinase and MITF were determined by Western-blotting 24 h after the irradiation. Bar graph represents mean ± SD from three independent experiments. * *p* < 0.05, ** *p* < 0.01 vs. control.

The Western blot analysis (Figure 6) indicated that lomefloxacin and moxifloxacin decreased the protein level of tyrosinase by about 55% (*p* < 0.0001) and 15% (*p* = 0.0384), respectively, compared with the control. The UVA radiation caused no significant increase in tyrosinase protein expression, whereas in melanocytes exposed to UVA radiation and fluoroquinolones level of tyrosinase protein was decreased by approximately 23% (*p* = 0.0009) and 14% (*p* = 0.0485), respectively, in cells treated with lomefloxacin and moxifloxacin.

**Figure 7:**
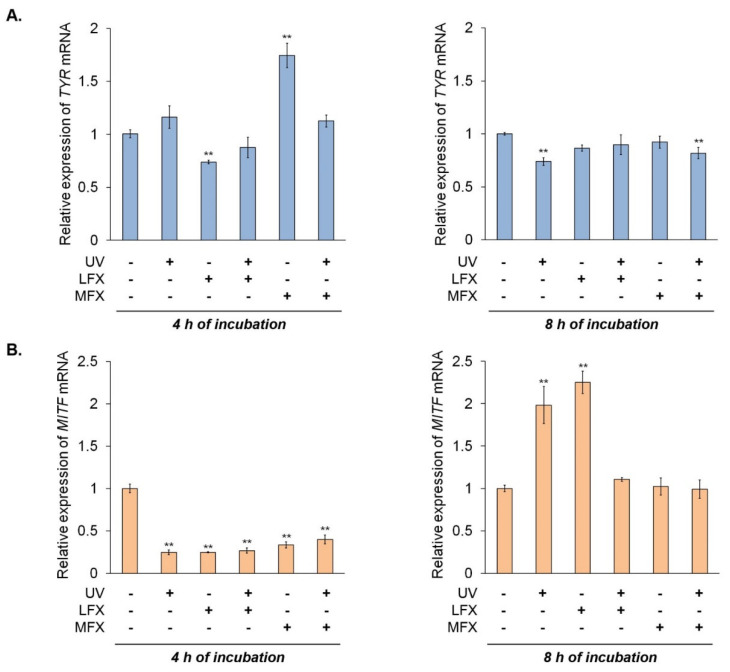
Fluoroquinolones alone and plus UVA radiation affect the expression of tyrosinase (*TYR*) and microphthalmia-associated transcription factor (*MITF*) at mRNA level in melanocytes. The cells were exposed to lomefloxacin (LFX) in concentration of 0.05 mM and moxifloxacin (MFX) in concentration of 0.1 mM. The mRNA levels of *TYR* (**A**) and *MITF* (**B**) were determined by RT-qPCR after 4 h and 8 h since the irradiation. Bar graph represents mean ± SD from three independent experiments. ** *p* < 0.01 vs. control.

The RT-qPCR analysis 4 h after the irradiation (Figure 7A) revealed that lomefloxacin inhibited the expression of tyrosinase mRNA by 0.27. Unexpectedly, we observed a significant increase of tyrosinase mRNA level by 0.74 in melanocytes treated with moxifloxacin. The simultaneous exposure to UVA radiation and fluoroquinolones had no significant effect on the mRNA level of tyrosinase. In the case of RT-qPCR analysis after 8 h since the irradiation, we noticed a decreased level of tyrosinase mRNA in melanocytes exposed to UVA radiation only (by 0.26) and UVA radiation plus moxifloxacin (by 0.18).

It was observed that MITF protein levels varied in the same manner as the expression of tyrosinase protein. Fluoroquinolones, both alone and with UVA radiation, caused a reduction of MITF protein level. Lomefloxacin and moxifloxacin alone decreased the protein level of MITF by 23% (*p* = 0.0006) and 44% (*p* < 0.0001), respectively, whereas drugs with UVA radiation caused a reduction of MITF protein level by about 13% (*p* = 0.0148) and 41% (*p* < 0.0001), respectively. UVA radiation alone increased MITF expression by 29% (*p* < 0.0001).

We observed very low expression of *MITF* in all samples 4 h after the irradiation (Figure 7B). However, 8 h after the irradiation of melanocytes, expression of *MITF* mRNA corresponded to changes observed in Western blot analysis. Irradiation of melanocytes resulted in an increase in *MITF* mRNA level by 0.98. Moxifloxacin alone and simultaneous exposure to UVA radiation and both fluoroquinolones did not significantly influence the expression of *MITF* mRNA. Unexpectedly, we observed high expression of *MITF* mRNA in cells treated with lomefloxacin—increased by 1.25 compared with the control.

## 4. Discussion

Drug-induced photosensitivity is the abnormal cutaneous response to exposure to the photosensitive drug and ultraviolet radiation. There are two types of photosensitivity reactions i.e., photoallergy and phototoxicity. The photoallergy is a cell-mediated hypersensitivity generated by a photoproduct acting as a hapten or as a complete antigen. The phototoxic reaction is the result of direct tissue/cellular damage by a photoproduct. FQs mainly cause phototoxic reactions, but photoallergy to these antibiotics also was reported [14,27]. Clinical manifestations of phototoxicity to FQs range from mild erythema of sun-exposed areas [14] to severe skin reactions, including bullous eruptions, hyperpigmented brown–gray patches [28], nail pigmentation [29], or photo-induced Stevens–Johnson syndrome [30].

Hyperpigmentation, one of the symptoms of FQs phototoxicity, typically is caused by an increase in melanin deposition in the basal and suprabasal layers of the skin [31]. Melanins are large biopolymers derived by the oxidation and polymerization of tyrosine [32]. The synthesis of melanins, termed melanogenesis, takes place in specialized membrane-bound organelles of melanocytes called melanosomes [33]. The first and rate-limiting step in melanogenesis is the oxidation of l-tyrosine to l-dihydroxyphenylalanine (l-DOPA) by tyrosinase. l-DOPA is rapidly subsequently oxidized to DOPAquinone, reactive intermediate and precursor to both eumelanin and pheomelanin. In the presence of sulfhydryl compounds, such as cysteine, DOPAquinone is converted to thiol adducts, 3- or 5-cysteinylDOPAs. The thiol adducts are then oxidized to 1,4-benzothiazine intermediates, which polymerize to give rise to reddish–yellow pheomelanin. The depletion of sulfhydryl compounds enhances eumelanin production. DOPAquinone undergoes intramolecular cyclization to cyclodopa, which reacts with unchanged DOPAquinone, yielding orange DOPAchrome. Then DOPAchrome is converted to 5,6-dihydroxyindole (DHI) and DHI-2-carboxylic acid (DHICA), which are subsequently oxidized and polymerized to form eumelanin [21,33,34].

Melanogenesis is a complex process, regulated by intrinsic and extrinsic factors. Intrinsic stimulators of melanin synthesis include hormones, eicosanoids, histamine, and endothelins. The dominant hormonal factor, which positively regulates melanogenesis, is a melanocyte-stimulating hormone (MSH). It induces expression and activity of melanogenesis-related proteins by increasing the level of intracellular cyclic adenosine monophosphate (cAMP) and activation of protein kinase-C (PKC). Other important stimulators of pigmentation are adrenocorticotropic hormone (ACTH) and estrogens; they enhance tyrosinase activity [35].

Melanins, especially eumelanin, have several biological functions. They provide protection against UV radiation and neutralize reactive oxygen species. Melanins possess the capacity to bind a heterogeneity of xenobiotics also. Drugs with the melanin affinity may be accumulated and retained in pigmented tissues, which has dual effects. On the one hand the drug-melanin complex acts as a depot system, from which the drug is slowly released in non-toxic concentrations. Due to this phenomenon, melanin may protect tissues against the harmful effects of xenobiotics. On the other hand, the long-exposure to the drug can contribute to a significant disturbance of homeostasis of melanin-containing cells and induction of a range of adverse effects on pigmented tissues [33,36,37].

Previously, we demonstrated that both lomefloxacin and moxifloxacin bound to melanin pigment, but had different affinities for this biopolymer. The total number of binding sites for lomefloxacin was 0.92 μmol/mg, whereas for moxifloxacin was 0.39 μmol/mg. These results indicated that both FQs may accumulate in melanin-containing cells and affect their cellular processes; however, not in the same manner [20,24]. We showed that lomefloxacin and moxifloxacin, either alone or plus UVA radiation, induced oxidative stress in melanocytes, but they had different effects on the activity and expression of antioxidant enzymes [26].

Moreover, it was reported earlier that FQs, either alone or activated by UVA radiation, affected melanogenesis [24,38,39,40,41]. Therefore, the aim of our study was the evaluation of the phototoxic effect of moxifloxacin and lomefloxacin on normal human melanocytes in terms of impact on melanin synthesis.

We observed significant alterations in melanin content, *MITF* expression, and tyrosinase activity and expression in melanocytes exposed to UVA radiation and/or tested FQs. Moreover, FQs affected the morphological features of melanocytes. It is interesting to note that changes in cell morphology were observed for drugs only in the highest tested concentrations, whereas biochemical and molecular alterations were induced by FQs in the entire range of tested concentrations. It is consistent with our previous studies. We demonstrated that exposure to FQs was connected with significant alterations of antioxidant enzymes activity despite no change in cell viability [26].

Among the extrinsic factors regulating melanogenesis, UV radiation is considered the most important, because it induces pigmentation in various ways [33,34]. Irradiation by UVA caused stimulation of MSH and ACTH. Moreover, under UVA radiation, NO production occurs in melanocytes that leads to accumulation of the cyclic guanosine 3′,5′-monophosphate (cGMP), activation of MITF, and tyrosinase [42]. Our results indicated that UVA radiation enhanced the melanogenesis process in melanocytes through activation of tyrosinase and induction of MITF synthesis.

The obtained results indicated that lomefloxacin and moxifloxacin alone inhibit melanogenesis. FQs caused a decrease in melanin content that might be consequence of observed inhibition of tyrosinase and decreased protein level of tyrosinase and MITF in melanocytes. It is consistent with the results of our previous studies. We demonstrated, that FQs (lomefloxacin, ciprofloxacin, moxifloxacin) decreased melanin content and tyrosinase activity in cultured normal melanocytes [24,38,39,40].

The data of current studies confirm different phototoxic potentials of moxifloxacin and lomefloxacin. Our results showed that moxifloxacin did not potentiate the changes in the melanization process induced by UVA radiation. The simultaneous exposure to UVA radiation and moxifloxacin did not cause a significant increase in either melanin content or tyrosinase activity compared with exposure to UVA radiation alone. In turn, melanocytes treated with lomefloxacin and exposed to UVA radiation were shown to possess increased melanin content and tyrosinase activity than non-treated cells exposed to UVA radiation. The obtained results correspond to observations of other in vitro studies. Marrot et al. [41] demonstrated that exposure to UVA radiation and lomefloxacin stimulated the tyrosinase activity in Caucasian melanocytes. The various effects of lomefloxacin and moxifloxacin on melanin synthesis may be associated with a different chemical structure of these FQs derivatives. The structure of phototoxicity relationship of FQs has been demonstrated. Analogs with a methoxy group at C-8 position, such as moxifloxacin, have lower phototoxic potential than 8-halogenated FQs, such as lomefloxacin [15,16].

Tyrosinase is a copper-containing glycoenzyme essential in melanin synthesis. This glycoenzyme catalyzes two sequential reactions: hydroxylation of monophenols to ortho-diphenols i.e., l-tyrosine to l-DOPA and the oxidation of ortho-diphenols to quinones i.e., l-DOPA to DOPAquinone and 5,6- dihydroxyindole to 5,6-dihydroxyquinone. The regulation of enzyme activity includes control of tyrosinase mRNA synthesis, alternative splicing and post-translational modification of protein [43,44]. Moreover, l-tyrosine and l-DOPA act as regulators of tyrosinase function. They stimulate the maturation of tyrosine, translocation to melanosomes, and protect the enzyme from degradation. l-tyrosine increases also the expression of the melanocyte-stimulating hormone (MSH) receptor, which enhances the synthesis and activation of tyrosinase [35,45].

Iozumi and co-workers [46] have indicated that the level of tyrosinase activity is not determined by the quantity of enzyme protein. Authors analyzed tyrosinase activity and amount in 20 different melanocyte-cell stram cultures and they observed that all cells possessed a similar abundance of the enzyme, whereas they had a different activity of tyrosinase. These data are in agreement with our results. We observed that alterations in tyrosinase activity, especially in cells exposed to UVA radiation and phototoxic lomefloxacin, did not correspond to changes in the protein level of the enzyme. Despite the low amount of tyrosinase, the enzyme activity was high. The possible explanation for the observed phenomenon may be the activation of the enzyme via an oxidative stress-dependent mechanism. Previously, we demonstrated that exposure to lomefloxacin and UVA radiation caused great changes in activity and level of antioxidant enzymes, especially hydrogen peroxide-scavenging enzymes [26]. The noticed phenomenon may result from the overproduction of ROS, in particular hydrogen peroxide. It has been demonstrated that hydrogen peroxide induces the expression of melanogenesis-related genes, among others phenylalanine hydroxylase (PAH). PAH catalyzes the conversion of phenylalanine to L-tyrosine, which regulates tyrosinase activity [45,47]. Moreover, hydrogen peroxide may stimulate tyrosinase directly [48].

MITF, a member of the basic helix–loop–helix (bHLH) transcription factor family, up-regulates tyrosinase gene transcription by binding cis-acting elements, including M-box, initiator region (Inr), and tyrosinase distal elements (TDE) [49,50]. It has been reported that MITF provides a continuous expression of the tyrosinase gene under normal conditions and increased generation of tyrosinase mRNA in response to UV radiation and endocrine factors [51]. Moreover, a number of regulatory gene elements responsible for basal and enhanced tyrosinase transcription were demonstrated; therefore, the expression of tyrosinase may be controlled by different factors, not only by MITF [49,51].

The current Western-blot analysis indicated that the level of MITF protein corresponded to the level of tyrosinase in melanocytes exposed to UVA radiation and/or FQs. We observed that FQs alone and plus UVA radiation decreased the production of tyrosinase and MITF proteins. Surprisingly, RT-qPCR analysis showed that the expression of *MITF* increased 8 h after the irradiation, whereas the level of tyrosinase mRNA was increased 4 h after the irradiation, especially in melanocytes exposed to UVA radiation alone. This correlation might result from the activation of tyrosinase expression by transcription factors other than MITF. Obtained data can be used as a basis for further studies.

## 5. Conclusions

Our results confirm that phototoxic reactions connected with FQs treatment may result from disturbances of melanogenesis. We demonstrated that lomefloxacin and moxifloxacin alone inhibited melanin synthesis in normal melanocytes. The tested FQs decreased MITF protein level, tyrosinase activity, and protein level, which resulted in a reduction of melanin content.

The obtained results indicate that the differences in phototoxic potential between lomefloxacin and moxifloxacin may be associated with various impacts on melanogenesis under UVA irradiation. The lower phototoxic moxifloxacin did not augment UVA-induced melanin synthesis. In turn, lomefloxacin, the most phototoxic FQ, potentiated UVA-induced activation of tyrosinase and melanin formation. However, the rise of tyrosinase activity in melanocytes exposed to lomefloxacin and UVA radiation was not correlated with an increase in expression of neither tyrosinase nor MITF.

The current studies show that the accurate analysis of the impact of drugs on melanogenesis at the cellular level requires evaluation of morphological, biochemical, and molecular changes. In the case of the influence of FQs on melanogenesis, we demonstrated for the first time that the phototoxic potential of lomefloxacin results from the direct enhancement of tyrosinase activity, without increasing enzyme expression. Moreover, our results indicate that biochemical and molecular changes in melanocytes induced by FQs and/or UVA radiation is not always accompanied by alterations of morphological features of cells, especially in case of exposure to low concentrations of drugs.

## Figures and Tables

**Table 1:** Nucleotide sequences of primers used in RT-qPCR analysis.

Gene	Forward Primer (5′→3′)	Reverse Primer (5′→3′)
GAPDH	CTTTTGCGTCGCCAG	TTGATGGCAACAATATCCAC
TYR	CAACAGCCATCAGTCTTTATG	CCTTCCAGTGTATTTCTAAAGC
MITF	CAGTACCTTTCTACCACTTTAG	CCTCTTTTTCACAGTTGGAG

## Data Availability

The data that support the findings of this study are available from the corresponding author upon reasonable request.
